# Prognostic Accuracy of Early Warning Scores for Clinical Deterioration in Patients With COVID-19

**DOI:** 10.3389/fmed.2020.624255

**Published:** 2021-02-01

**Authors:** Ying Su, Min-jie Ju, Rong-cheng Xie, Shen-ji Yu, Ji-li Zheng, Guo-guang Ma, Kai Liu, Jie-fei Ma, Kai-huan Yu, Guo-wei Tu, Zhe Luo

**Affiliations:** ^1^Department of Critical Care Medicine, Zhongshan Hospital, Fudan University, Shanghai, China; ^2^Department of Critical Care Medicine, Xiamen Branch, Zhongshan Hospital, Fudan University, Xiamen, China; ^3^Department of Nursing, Zhongshan Hospital, Fudan University, Shanghai, China; ^4^Department of Hepatobiliary Surgery, Remin Hospital of Wuhan University, Wuhan, China

**Keywords:** COVID-19, community-acquired pneumonia, early warning score, NEWS, NEWS 2, NEWS-C, quick sequential organ failure assessment

## Abstract

**Background:** Early Warning Scores (EWS), including the National Early Warning Score 2 (NEWS2) and Modified NEWS (NEWS-C), have been recommended for triage decision in patients with COVID-19. However, the effectiveness of these EWS in COVID-19 has not been fully validated. The study aimed to investigate the predictive value of EWS to detect clinical deterioration in patients with COVID-19.

**Methods:** Between February 7, 2020 and February 17, 2020, patients confirmed with COVID-19 were screened for this study. The outcomes were early deterioration of respiratory function (EDRF) and need for intensive respiratory support (IRS) during the treatment process. The EDRF was defined as changes in the respiratory component of the sequential organ failure assessment (SOFA) score at day 3 (ΔSOFA_resp_ = SOFA _resp_ at day 3–SOFA_resp_ on admission), in which the positive value reflects clinical deterioration. The IRS was defined as the use of high flow nasal cannula oxygen therapy, noninvasive or invasive mechanical ventilation. The performances of EWS including NEWS, NEWS 2, NEWS-C, Modified Early Warning Scores (MEWS), Hamilton Early Warning Scores (HEWS), and quick sepsis-related organ failure assessment (qSOFA) for predicting EDRF and IRS were compared using the area under the receiver operating characteristic curve (AUROC).

**Results:** A total of 116 patients were included in this study. Of them, 27 patients (23.3%) developed EDRF and 24 patients (20.7%) required IRS. Among these EWS, NEWS-C was the most accurate scoring system for predicting EDRF [AUROC 0.79 (95% CI, 0.69–0.89)] and IRS [AUROC 0.89 (95% CI, 0.82–0.96)], while NEWS 2 had the lowest accuracy in predicting EDRF [AUROC 0.59 (95% CI, 0.46–0.720)] and IRS [AUROC 0.69 (95% CI, 0.57–0.81)]. A NEWS-C ≥ 9 had a sensitivity of 59.3% and a specificity of 85.4% for predicting EDRF. For predicting IRS, a NEWS-C ≥ 9 had a sensitivity of 75% and a specificity of 88%.

**Conclusions:** The NEWS-C was the most accurate scoring system among common EWS to identify patients with COVID-19 at risk for EDRF and need for IRS. The NEWS-C could be recommended as an early triage tool for patients with COVID-19.

## Introduction

The outbreak of coronavirus disease 2019 (COVID-19) has recently become a public health emergency of international concern (1). A novel coronavirus, termed as severe acute respiratory syndrome coronavirus 2 (SARS-CoV-2), was isolated as the pathogen of COVID-19 (2). As of October 18, 2020, there have been more than 40 million confirmed COVID-19 cases and 1.1 million deaths globally from World Health Organization reports.

With a sharp increase in the number of cases and limited medical resources, healthcare systems worldwide are facing unprecedented challenges (3). Although the majority of patients with COVID-19 have mild symptoms, patients with advanced age and chronic comorbidities such as hypertension tend to have poor outcomes (4, 5). Patients infected with SARS-CoV-2 tend to get worse from illness onset with a median duration of 7 days, in which severe type may deteriorate to acute respiratory distress syndrome (ARDS) or multiple organ failure (6, 7). During the outbreak of the COVID-19 crisis, early and quick recognition of patients who are at high risk of clinical deterioration would therefore be significantly important (8). A severity-based approach is urgently needed for triaging high risk patients with COVID-19 (9).

The Early Warning Scores (EWS) are a variety of physiologic scoring systems widely used in the world. These systems are based on bedside indices that can be obtained easily and rapidly such as heart rate, respiratory rate, systolic blood pressure, and peripheral oxygen saturation (SpO_2_), allowing quick and accurate identify patients at high risk of clinical deterioration. Now, various EWS were developed for early recognition of clinical deterioration. The National Early Warning Score (NEWS), the most common EWS, was initially recommended by the Royal College of Physicians (RCP) (10). It has been proved that NEWS was associated with ICU admission and death outside ICU (11, 12). Its updated version, the National Early Warning Score 2 (NEWS2), with a new SpO_2_ scoring scale, was published by the RCP in 2017 to improve prediction for clinical deterioration in patients with a hypercapnic respiratory failure (13). Other versions of EWS such as the Modified Early Warning Score (MEWS) (14, 15) and Hamilton Early Warning Score (HEWS) (16) have also been recently developed to improve the early recognition of hospitalized patients at risk for deterioration, with a significant degree of variation in the clinical variables and the weightings assigned.

Currently, guidelines from the RCP recommends the use of the NEWS2 for initial assessment in patients with COVID-19 (17). Moreover, NEWS-C, a new version of modified NEWS, has also been recommended for triage decisions in patients with COVID-19 (18, 19). However, the recommendations were only based on expert opinions and have not been fully validated in COVID-19 patients.

In this study, we aimed to compare the performance of EWS including NEWS, NEWS2, NEWS-C, HEWS, MEWS, and quick sepsis-related organ failure assessment (qSOFA) to predict early deterioration of respiratory function (EDRF) and the need for intensive respiratory support (IRS) in patients with COVID-19.

## Materials and Methods

### Study Design and Participants

The study was approved by the Ethics Commission of Renmin Hospital of Wuhan University (WDRY2020-K048) and was conducted in accordance with the amended Declaration of Helsinki. Written informed consent was waived by the Ethics Commission in the setting of COVID-19 crisis in Wuhan.

Patients with age ≥18 years and confirmed COVID-19 admitted between February 7, 2020 and February 17, 2020 were screened in our study. We excluded patients for pregnancy, death within 48 h of admission, or having a Do Not Resuscitate order. COVID-19 was diagnosed by the real-time RT-PCR method on nasal or pharyngeal swab specimens.

### Date Collection

Baseline demographics, clinical characteristics on hospital arrival including symptoms, vital signs, and oxygen therapy, laboratory findings, treatments, and outcomes were prospectively collected by two trained reviewers. The NEWS, NEWS-C, NEWS2, HEWS, MEWS, and qSOFA were calculated based on the demographic and clinical characteristics of each patient (Supplementary Table 1).

### Outcome Assessment

Respiratory function was assessed according to the respiratory component of the sequential organ failure assessment score (SOFA_resp_). The EDRF was defined as a positive change in respiratory function at day 3 (ΔSOFA_resp_ = SOFA_resp_ at day 3–SOFA_resp_ on admission). The positive value of ΔSOFA_resp_ reflects clinical deterioration. The IRS was defined as the use of high flow nasal cannula oxygen therapy, noninvasive or invasive mechanical ventilation. IRS was considered if the patients met the following criteria: a respiratory rate of ≥30 breaths per minute and arterial oxygen saturation (SaO_2_) ≤ 93% or a ratio of the partial pressure of arterial oxygen (PaO_2_) to inspired oxygen (FiO_2_) of 300 mmHg or less while the patient was receiving oxygen therapy of ≥10 L/min for at least 15 min. The choices of respiratory support method (high flow nasal cannula oxygen therapy, noninvasive or invasive mechanical ventilation) were at the discretion of the attending clinicians.

### Statistical Analysis

Data distribution was evaluated using the Kolmogorov-Smirnov test. Continuous variables were expressed as mean ± standard deviation or median interquartile range as appropriate. Categorical variables were expressed as frequencies and percentages. Baseline data were compared using the Student's *t-*test or the Mann-Whitney U test for continuous variables and the chi-square test or Fisher's exact test for categorical variables. Receiver operating characteristic (ROC) curves were constructed to assess the performance of EWS, and the optimal cut-off values were calculated by the Youden index. The area under the receiver operating characteristic curve (AUROC) were compared by the method described by Hanley and McNeil (20). All statistical analyses were performed using the SPSS software package, version 13.0 (SPSS, Inc., Chicago, IL, USA) and MedCalc software 15.0 (MedCalc Software Ltd, Ostend, Belgium). A two-tailed *P* value of <0.05 was considered statistically significant.

## Results

### Patient Characteristics

Between February 7, 2020 and February 17, 2020, a total of 123 patients with COVID-19 were screened for inclusion. Of these patients, seven patients were excluded, including one patient with pregnancy, three patients who died within 48 h after admission, and three patients with DNR order. Finally, 116 patients were included for this study.

**Figure 1:**
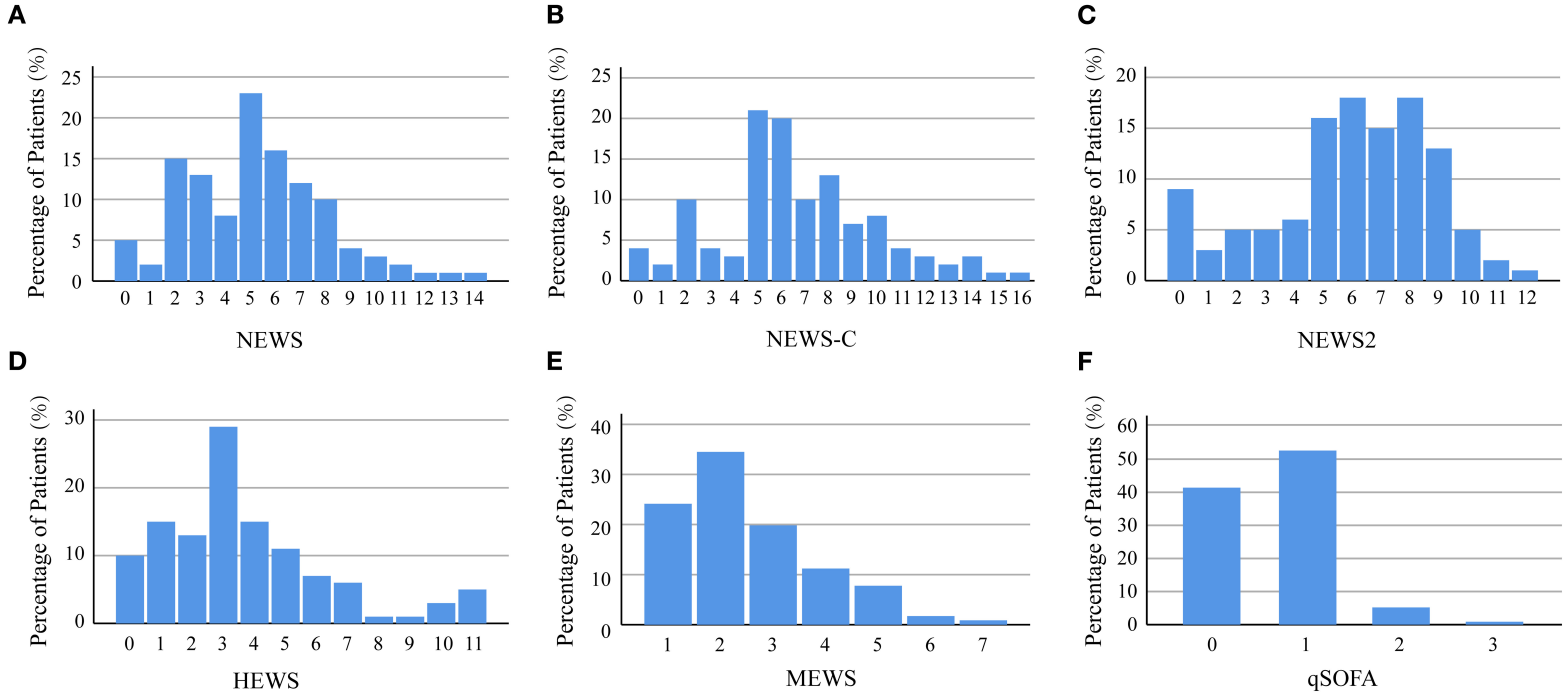
Distribution of patients by NEWS **(A)**, NEWS-C **(B)**, NEWS2 **(C)**, HEWS **(D)**, MEWS **(E)**, and qSOFA **(F)**. HEWS, Hamilton Early Warning Score; MEWS, Modified Early Warning Score; NEWS, National Early Warning Score; NEWS-C, modified NEWS; NEWS 2, National Early Warning Score 2; qSOFA, quick Sepsis-related Organ Failure Assessment.

The baseline characteristics were shown in Table 1. Of 116 patients, the median age was 63 [IQR 51, 72] years and 47.4% were men. Fever was the most common symptom (86.2%), followed by fatigue (85.3%), cough (69.0%), and dyspnea (56.9%). The baseline NEWS, NEWS-C, NEWS2, HEWS, MEWS, and qSOFA at admission were 5 [3, 7], 6 [5, 9], 6 [5, 8], 3 [2, 5], 2 [2, 3], and 1 [0, 1], respectively. The distributions of all patients by NEWS, NEWS-C, NEWS2, HEWS, MEWS, and qSOFA at admission were presented in Figure 1.

**Table 1:** Clinical characteristics of COVID-19 patients.

	**Entire cohort**	**No EDRF**	**EDRF**	***P* value**	**No need for IRS**	**Need for IRS**	***P* value**
Number of patients	116	89	27		92	24	
Age (years)	63 [51, 72]	61 [49, 69]	71 [64, 80]	<0.001	61 [48, 69]	73 [65, 81]	<0.001
Gender (male), *n* (%)	55 (47.4)	42 (47.2)	13 (48.1)	1.00	42 (45.7)	13 (54.2)	0.50
Smoking history, *n* (%)	10 (8.6)	8 (9.0)	2 (7.4)	1.00	9 (9.8)	1 (4.2)	0.69
**Comorbidities**							
Hypertension, *n* (%)	38 (32.8)	22 (24.7)	16 (59.3)	0.002	25 (27.2)	13 (54.2)	0.02
Diabetes mellitus, *n* (%)	20 (17.2)	16 (18.0)	4 (14.8)	1.00	15 (16.3)	5 (20.8)	0.56
CAD, *n* (%)	12 (10.3)	9 (10.1)	3 (11.1)	1.00	9 (9.8)	3 (12.5)	0.71
COPD, *n* (%)	2 (1.7)	2 (2.2)	0 (0)	1.00	0 (0)	2 (8.3)	0.04
Cerebrovascular disease, *n* (%)	2 (1.7)	0 (0)	2 (7.4)	0.05	1 (1.1)	1 (4.2)	0.37
Chronic renal disease, *n* (%)	4 (3.4)	3 (3.4)	1 (3.7)	1.00	3 (3.3)	1 (4.2)	1.00
**Signs and symptoms**							
Fever, *n* (%)	100 (86.2)	75 (84.3)	25 (92.6)	0.35	77 (83.7)	23 (95.8)	0.19
Cough, *n* (%)	80 (69.0)	58 (65.2)	22 (81.5)	0.15	62 (67.4)	18 (75)	0.62
Sputum production, *n* (%)	15 (12.9)	9 (10.1)	6 (22.2)	0.11	11 (12)	4 (16.7)	0.51
Fatigue, *n* (%)	99 (85.3)	73 (82.0)	26 (96.3)	0.12	77 (83.7)	22 (91.7)	0.52
Headache, *n* (%)	6 (5.2)	6 (6.7)	0 (0)	0.33	6 (6.65)	0 (0)	0.34
Dyspnea, *n* (%)	66 (56.9)	42 (47.2)	24 (88.9)	<0.001	44 (47.8)	22 (91.7)	<0.001
Nausea or vomiting, *n* (%)	25 (21.6)	17 (19.1)	8 (29.6)	0.29	19 (20.7)	6 (25)	0.78
Diarrhea, *n* (%)	23 (19.8)	18 (20.2)	5 (18.5)	1.00	19 (20.7)	4 (16.7)	0.78
Anorexia, *n* (%)	8 (6.9)	3 (3.4)	5 (18.5)	0.02	2 (2.2)	6 (25)	<0.001
Myalgia or arthralgia, *n* (%)	10 (8.6)	8 (9.0)	2 (7.4)	1.00	8 (8.7)	2 (8.3)	1.00
Onset of symptom to hospital admission, days	12 [9, 16]	12 [9, 18]	9 [6, 13]	0.02	12 [9, 17]	10 [7, 16]	0.16
**Vital signs at hospital admission**							
Altered mental status, *n* (%)	6 (5.2)	1 (1.1)	5 (18.5)	0.003	0 (0)	6 (25)	<0.001
Heart rate, beats/minute	90 [79, 102]	89 [79, 101]	96 [83, 106]	0.09	88 [78, 101]	95 [86, 107]	0.02
Respiratory rate, breaths/minute	23 [20, 29]	22 [20, 26]	28 [21, 34]	<0.01	22 [20, 25]	32 [22, 35]	<0.001
Systolic blood pressure, mm Hg	132 [122, 145]	131 [122, 144]	141 [127, 151]	0.19	131 [122, 144]	139 [122, 152]	0.25
Diastolic blood pressure, mm Hg	78 [68, 84]	78 [69, 83]	77 [66, 93]	0.52	79 [69, 84]	75 [66, 92]	0.96
**Early warning scores at hospital admission**							
NEWS	5 [3, 7]	5 [3, 6]	7 [5, 9]	<0.001	5 [3, 6]	8 [6, 10]	<0.001
NEWS-C	6 [5, 9]	6 [5, 8]	10 [6, 12]	<0.001	6 [4, 8]	10 [8, 13]	<0.001
NEWS 2	6 [5, 8]	6 [5, 8]	7 [4, 9]	0.16	6 [4, 8]	7 [6, 9]	0.004
HEWS	3 [2, 5]	3 [2, 4]	6 [3, 9]	0.001	3 [1, 4]	7 [5, 10]	<0.001
MEWS	2 [2, 3]	2 [1, 3]	3 [2, 5]	0.001	2 [1, 3]	4 [3, 5]	<0.001
qSOFA	1 [0, 1]	1 [0, 1]	1 [0, 1]	0.03	1 [0, 1]	1 [1, 2]	<0.001
**Respiratory status assessment**							
PaO_2_ on admission, mmHg	90 [69, 95]	92 [78, 96]	69 [55, 88]	<0.001	92 [77, 96]	69 [55, 83]	<0.001
PaCO_2_ on admission, mmHg	41 [38, 44]	42 [39, 45]	39 [37, 44]	0.11	41 [38, 45]	41 [38, 44]	0.62
PaO_2_/FiO_2_ on admission, mmHg	292 [245, 326]	305 [272, 328]	245 [167, 303]	0.001	305 [274, 329]	196 [150, 260]	<0.001
SOFA_resp_ on admission	2 [1, 2]	1 [1, 2]	2 [1, 3]	0.004	1 [1, 2]	3 [2, 3]	<0.001
PaO_2_ at day 3, mmHg	94 [85, 96]	95 [92, 97]	63 [52, 84]	<0.001	95 [92, 97]	63 [51, 80]	<0.001
PaCO_2_ at day 3, mmHg	41 [37, 46]	42 [38, 46]	39 [36, 45]	0.10	41 [37, 46]	42 [38, 46]	0.85
PaO_2_/FiO_2_ at day 3, mmHg	314 [241, 330]	326 [277, 349]	156 [87, 197]	<0.001	326 [277, 334]	102 [74, 159]	<0.001
SOFA_resp_ at day 3	1 [1, 2]	1 [1, 2]	3 [3, 4]	<0.001	1 [1, 2]	3 [3, 4]	<0.001
EDRF, *n* (%)	27 (23.3)	0 (0)	27 (100)	<0.001	7 (7.6)	20 (83.3)	<0.001
Need for IRS, *n* (%)	24 (20.7)	4 (4.5)	20 (74.1)	<0.001	0 (0)	24 (100)	<0.001
**Respiratory support**							
High flow nasal cannula, *n* (%)	24 (20.7)	4 (4.5)	20 (74.1)	<0.001	0 (0)	24 (100)	<0.001
Non-invasive mechanical ventilation, *n* (%)	5 (4.3)	0 (0)	5 (18.5)	<0.001	0 (0)	5 (20.8)	<0.001
Invasive mechanical ventilation, *n* (%)	8 (6.9)	1 (1.1)	7 (25.9)	<0.001	0 (0)	8 (33.3)	<0.001
Need for vasopressor support, *n* (%)	9 (7.8)	2 (2.2)	7 (25.9)	<0.001	1 (1.1)	8 (33.3)	<0.001
Renal replacement therapy, *n* (%)	3 (2.6)	2 (2.2)	1 (3.7)	0.55	2 (2.2)	1 (4.2)	0.50
Extracorporeal membrane oxygenation, *n* (%)	1 (0.9)	0 (0)	1 (3.7)	0.23	0 (0)	1 (4.2)	0.21
Hospital mortality, *n* (%)	9 (7.8)	1 (1.1)	8 (29.6)	<0.001	0 (0)	9 (37.5)	<0.001
Hospital length of stay, days	29 [18, 36]	28 [18, 34]	38 [8, 49]	0.04	29 [18, 35]	37 [7, 49]	0.28

*Continuous variables are shown as the mean ± SD or median [IQR], as appropriate. Categorical variables are shown as number (%)*.

*COVID-19, coronavirus disease 2019; CAD, coronary artery disease; COPD, chronic obstructive pulmonary disease; FiO_2_, fraction of inspired oxygen; PaO_2_, partial pressure of oxygen; HEWS, Hamilton Early Warning Score; MEWS, Modified Early Warning Score; NEWS, National Early Warning Score; NEWS-C, modified NEWS; NEWS 2, National Early Warning Score 2; qSOFA, quick Sepsis-related Organ Failure Assessment; EDRF, early deterioration of respiratory function; IRS, intensive respiratory support*.

The baseline PaO_2_/FiO_2_ on admission was 292 [245, 326] mmHg. At day 3, the median PaO_2_/FiO_2_ was 314 [241, 330] mmHg. A total of 27 (23.3%) patients developed EDRF according to the ΔSOFA_resp_ (SOFA_resp_ at day 3–baseline SOFA_resp_), in which a positive value reflected clinical deterioration. Patients with EDRF tended to be older and had a higher rate of hypertension than those without EDRF (all *P* < 0.01). Compared with the patients without EDRF, the patients with EDRF have higher proportions of dyspnea (88.9 vs. 47.2%; *P* < 0.001) and higher respiratory rate [28 (21, 22) vs. 22 (20, 23) breaths/minute; *P* < 0.01] but lower baseline PaO_2_/FiO_2_ value [245 (167, 303) vs. 305 (272, 328) mmHg; *P* < 0.001]. On admission, patients with EDRF had higher NEWS, NEWS-C, HEWS, MEWS, and qSOFA than non-EDRF patients (all *P* < 0.05; Table 1). However, the NEWS2 between patients with EDRF and non-EDRF was comparable (*P* = 0.16; Table 1).

A total of 24 patients (20.7%) needed IRS during the period of hospital stay. Patients with IRS also tended to be older and had a higher rate of hypertension than those without IRS (all *P* < 0.05). Compared with the patients without IRS, the patients requiring IRS have higher proportions of dyspnea (91.7 vs. 47.8%; *P* < 0.001) and a higher respiratory rate [32 (24, 25) vs. 22 (20, 26); *P* < 0.001] but a lower baseline PaO_2_/FiO_2_ value [196 (150, 260) vs. 305 (274, 329); *P* < 0.001]. Patients with IRS had a higher NEWS, NEWS-C, NEWS2, HEWS, MEWS, and qSOFA than non-IRS patients (all *P* < 0.001; Table 1). A total of 20 patients (17.2%) developed both IRS and EDRF in this cohort. The hospital mortality rate was 7.8%. The mortality in patients with EDRF or IRS was higher than those without EDRF or IRS (all *P* < 0.001).

### Performance of EWS for Clinical Deterioration

**Figure 2:**
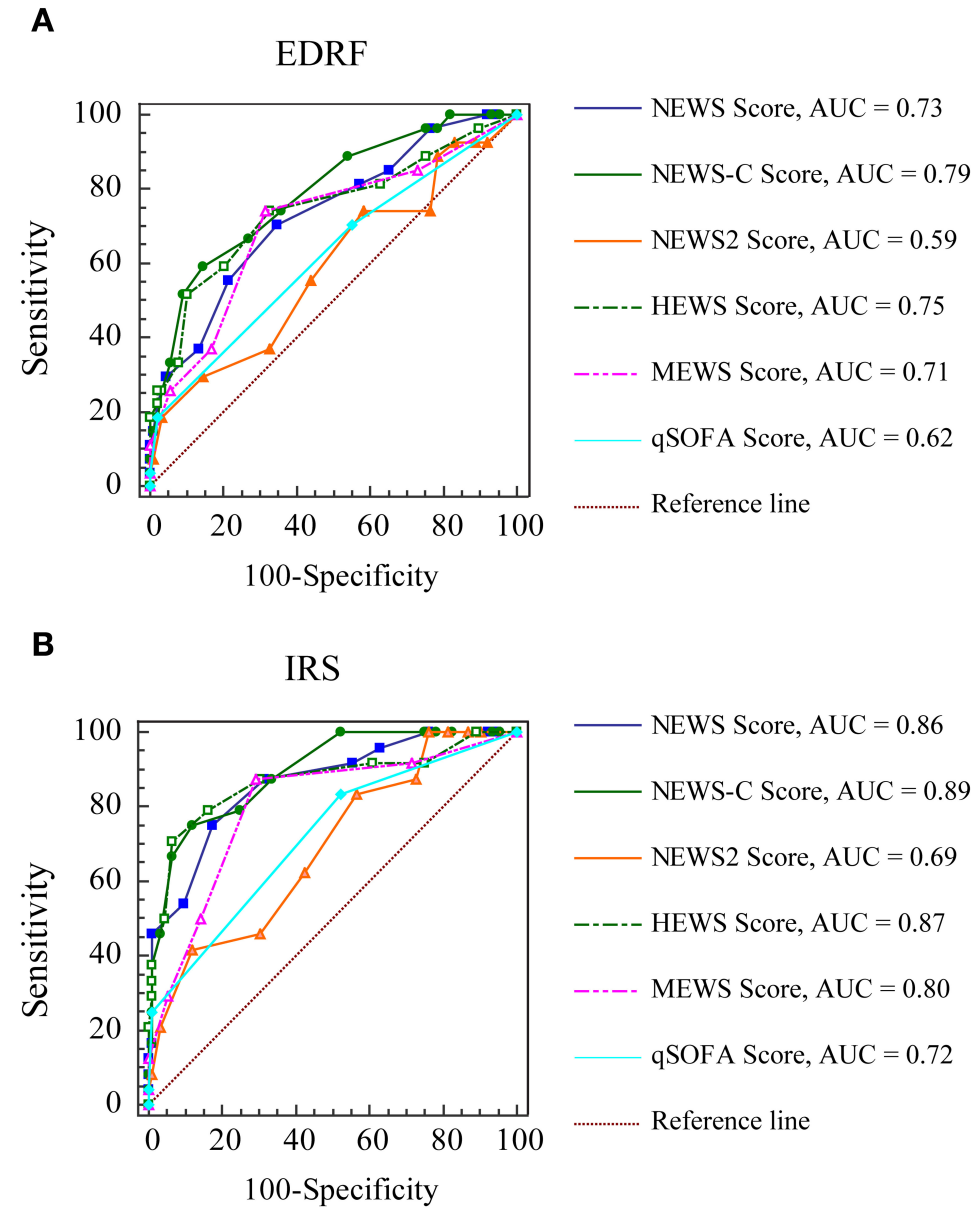
The receiver operating characteristic curves of early warning scores for clinical deterioration. **(A)** EDRF; **(B)** IRS. AUC, area under the curve; HEWS, Hamilton Early Warning Score; MEWS, Modified Early Warning Score; NEWS, National Early Warning Score; NEWS-C, modified NEWS; NEWS 2, National Early Warning Score 2; qSOFA, quick Sepsis-related Organ Failure Assessment; EDRF, early deterioration of respiratory function; IRS, intensive respiratory support.

To assess the utility of EWS to predict EDRF and need for IRS, the ROC curves were constructed and the AUROCs were calculated (Figure 2). Table 2 listed AUROC, optimal cutoff value, sensitivity, specificity, and positive and negative predictive values of EWS.

**Table 2:** Performance of early warning scores in predicting clinical deterioration.

**Outcomes**	**Predictors**	**AUROC**	**95% CI**	**P**	**Cut-off**	**Sensitivity (%)**	**Specificity (%)**	**PPV**	**NPV**	**LR+**	**LR–**
EDRF	NEWS	0.73 ± 0.06	0.62–0.84	<0.001	5	81.5	42.7	30.1	88.4	1.42	0.43
					**6**	**70.4**	**65.2**	**38**	**87.9**	**2.02**	**0.45**
					7	55.6	78.7	44.1	85.4	2.6	0.57
	NEWS-C	0.79 ± 0.05	0.69–0.89	<0.001	8	66.7	73.0	42.9	87.8	2.47	0.46
					**9**	**59.3**	**85.4**	**55.2**	**87.4**	**4.06**	**0.48**
					10	51.9	91.0	63.6	86.2	5.77	0.53
	NEWS2	0.59 ± 0.07	0.46–0.72	0.18	5	74.1	23.6	22.7	75	0.97	1.1
					**6**	**74.1**	**41.6**	**27.8**	**84.1**	**1.27**	**0.62**
					7	55.6	56.2	27.8	80.6	1.27	0.79
	HEWS	0.75 ± 0.06	0.63–0.86	<0.001	5	59.3	79.8	47.1	86.6	2.93	0.51
					**6**	**51.9**	**89.9**	**60.9**	**86.0**	**5.13**	**0.54**
					7	33.3	92.1	56.2	82	4.24	0.72
	MEWS	0.71 ± 0.06	0.59–0.83	<0.001	2	85.2	27.0	26.1	85.7	1.17	0.55
					**3**	**74.1**	**68.5**	**41.7**	**89.7**	**2.35**	**0.38**
					4	37.0	83.2	40	81.3	2.2	0.76
	qSOFA	0.62 ± 0.06	0.51–0.74	0.04	1	70.4	44.9	27.9	83.3	1.28	0.66
					**2**	**18.5**	**97.8**	**71.4**	**79.8**	**8.24**	**0.83**
Need for IRS	NEWS	0.86 ± 0.04	0.78–0.95	<0.001	6	87.5	68.5	42	95.5	2.78	0.18
					**7**	**75**	**82.6**	**52.9**	**92.7**	**4.31**	**0.3**
					8	54.2	90.2	59.1	88.3	5.54	0.51
	NEWS-C	0.89 ± 0.03	0.82–0.96	<0.001	8	79.2	75	45.2	93.2	3.17	0.28
					**9**	**75**	**88**	**62.1**	**93.1**	**6.27**	**0.28**
					10	66.7	93.5	72.7	91.5	10.22	0.36
	NEWS2	0.69 ± 0.06	0.57–0.81	0.002	8	45.8	69.6	28.2	83.1	1.51	0.78
					**9**	**41.7**	**88.0**	**47.6**	**85.3**	**3.48**	**0.66**
					10	20.8	96.7	62.5	82.4	6.39	0.82
	HEWS	0.87 ± 0.05	0.77–0.97	<0.001	5	79.2	83.7	55.9	93.9	4.86	0.25
					**6**	**70.8**	**93.5**	**73.9**	**92.5**	**10.86**	**0.31**
					7	50	95.7	75	88	11.5	0.52
	MEWS	0.80 ± 0.05	0.70–0.90	<0.001	2	91.7	28.3	25	92.9	1.28	0.29
					**3**	**87.5**	**70.7**	**43.7**	**95.6**	**2.98**	**0.18**
					4	50	85.9	48	86.8	3.54	0.58
	qSOFA	0.72 ± 0.05	0.61–0.82	<0.001	**1**	**83.3**	**47.8**	**29.4**	**91.7**	**1.6**	**0.35**
					2	25	98.9	85.7	83.5	23	0.76

*AUROC, area under the receiver operating characteristic curve; CI, confidence interval; LR, likelihood ratio; PPV, positive predictive value; NPV, negative predictive value; HEWS, Hamilton Early Warning Score; MEWS, Modified Early Warning Score; NEWS, National Early Warning Score; NEWS-C, modified NEWS; NEWS 2, National Early Warning Score 2; qSOFA, quick Sepsis-related Organ Failure Assessment; EDRF, early deterioration of respiratory function; IRS, intensive respiratory support. Bold: the optimal cut-off values according to Youden index*.

Among these EWS, NEWS-C was the most accurate scoring system for predicting EDRF {AUROC 0.79 [95% confidence interval (CI), 0.69–0.89]}. The AUROC of the NEWS-C for predicting EDRF was much higher than that for NEWS2 (0.59, 95% CI 0.46–0.72; *P* < 0.001) and qSOFA (0.62, 95% CI 0.51–0.74; *P* < 0.001). The AUROC of NEWS-C for predicting EDRF was also larger than NEWS (0.73, 95% CI 0.62–0.84), HEWS (0.75, 95% CI 0.63–0.86), and MEWS (0.71, 95% CI 0.59–0.83), although the difference is not statistically significant (Table 3). A NEWS-C ≥ 9 had a sensitivity of 59.3% and a specificity of 85.4% for predicting EDRF.

**Table 3:** The cross-comparisons between AUROCs of early warning scores for predicting EDRF.

**AUROC**		**NEWS**	**NEWS-C**	**NEWS2**	**HEMS**	**MEWS**	**qSOFA**
		**0.73** **[0.62–0.84]**	**0.79** **[0.69–0.89]**	**0.59** **[0.46–0.72]**	**0.75** **[0.63–0.86]**	**0.71** **[0.59–0.83]**	**0.62** **[0.51–0.74]**
NEWS	0.73 [0.62–0.84]	/	0.07	0.006	0.66	0.49	0.004
NEWS-C	0.79 [0.69–0.89]	0.07	/	<0.001	0.31	0.08	<0.001
NEWS2	0.59 [0.46–0.72]	0.006	<0.001	/	0.003	0.02	0.57
HEMS	0.75 [0.63–0.86]	0.66	0.31	0.003	/	0.17	0.008
MEWS	0.71 [0.59–0.83]	0.49	0.08	0.02	0.17	/	0.10
qSOFA	0.62 [0.51–0.74]	0.004	<0.001	0.57	0.008	0.10	/

*AUROC, area under the receiver operating characteristic curve; HEWS, Hamilton Early Warning Score; MEWS, Modified Early Warning Score; NEWS, National Early Warning Score; NEWS-C, modified NEWS; NEWS 2, National Early Warning Score 2; qSOFA, quick Sepsis-related Organ Failure Assessment; IRS, intensive respiratory support*.

Among these EWS, NEWS-C was the most accurate scoring system for predicting IRS [AUROC 0.89 (95% CI, 0.82–0.96)]. The AUROC of the NEWS-C for predicting IRS was much higher than that for NEWS2 (0.69, 95% CI 0.57–0.81; *P* < 0.001), MEWS (0.80, 95% CI 0.70–0.90; *P* = 0.03), and qSOFA (0.72, 95% CI 0.61–0.82; *P* < 0.001). The AUROC of NEWS-C for predicting IRS was also higher than NEWS (0.86, 95% CI 0.78–0.95) and HEWS (0.87, 95% CI 0.77–0.97), although the difference is not statistically significant (Table 4). For predicting IRS, a NEWS-C ≥ 9 had a sensitivity of 75% and a specificity of 88%.

**Table 4:** The cross-comparisons between AUROCs of Early Warning Scores for predicting IRS.

**AUROC**		**NEWS**	**NEWS-C**	**NEWS2**	**HEMS**	**MEWS**	**qSOFA**
		**0.86** **[0.78–0.95]**	**0.89** **[0.82–0.96]**	**0.69** **[0.57–0.81]**	**0.87** **[0.77–0.97]**	**0.80** **[0.70–0.90]**	**0.72** **[0.61–0.82]**
NEWS	0.86 [0.78–0.95]	/	0.28	0.001	0.82	0.049	<0.001
NEWS-C	0.89 [0.82–0.96]	0.28	/	<0.001	0.54	0.03	<0.001
NEWS2	0.69 [0.57–0.81]	0.001	<0.001	/		0.04	0.60
HEMS	0.87 [0.77–0.97]	0.82	0.54	0.003	/	0.01	0.001
MEWS	0.80 [0.70–0.90]	0.049	0.03	0.04		/	0.10
qSOFA	0.72 [0.61–0.82]	<0.001	<0.001	0.60	0.001	0.10	/

*AUROC, area under the receiver operating characteristic curve; HEWS, Hamilton Early Warning Score; MEWS, Modified Early Warning Score; NEWS, National Early Warning Score; NEWS-C, modified NEWS; NEWS 2, National Early Warning Score 2; qSOFA, quick Sepsis-related Organ Failure Assessment; IRS, intensive respiratory support*.

## Discussion

Until now, there are limited studies to evaluate the predictive value of EWS in patients with COVID-19. In the current study, we found that the NEWS-C was the most accurate scoring system among common EWS for predicting EDRF and IRS in patients with COVID-19. On the contrary, NEWS 2 had the lowest accuracy for predicting both outcomes.

EWS have been developed and widely used around the world for early recognition of clinical deterioration (21). The NEWS, endorsed by RCP, is already used for predicting deterioration in many hospitals across the United Kingdom (10). It is reported that the NEWS has better performance than other EWS to identify patients at risk of ICU admission and mortality (12). Moreover, the NEWS was more accurate in predicting clinical deterioration than qSOFA in infected patients outside the ICU (24).

The NEWS 2, updated version of NEWS, was recommended by RCP in 2017. The new SpO_2_ scoring scale in NEWS 2, with a lower SpO_2_ threshold than NEWS, was implemented to avoid over-use of supplemental oxygen and facilitate management in hypercapnic patients (27). Recently, the NEWS 2 has been recommended for predicting clinical deterioration in patients with COVID-19 (17). In our study, NEWS 2 had a lower performance than NEWS in predicting EDRF and IRS. This is in accordance with previous study, which demonstrated that NEWS 2 did not predict clinical outcome in elderly patients with COVID-19 (28). The possible reasons were as follows: (1) the incidence of type II respiratory failure in patients with COVID-19 was low in this study; and (2) the NEWS2 modifications to NEWS may not improve discrimination of poor outcome in hospital patients including those with type II respiratory failure (26). Therefore, NEWS 2 may be inappropriate for triage decision in patients with COVID-19.

A modified NEWS, termed NEWS-C, has also been recommended for triage decision in patients with COVID-19 (18, 19). The MEWS (14) and HEWS (16) have been developed to early identify clinical deterioration in generally hospitalized patients, with a significant degree of variation in the clinical variables and the weightings assigned. In this study, the NEWS-C had largest AUROC for predicting EDRF and IRS in these EWS. NEWS-C modifications to EWS added an age ≥65 years as an independent component. Several studies have showed that old aging was independently associated with mortality in patients with COVID-19 (4, 23). Therefore, it may offer better predictive performance than other EWS.

qSOFA, consisting of three clinical variables (mental status, respiratory rate, and blood pressure), has been proposed as a rapid screening tool for infected patients (29). The effectiveness of the qSOFA has been validated in various heterogeneous sepsis patients (30, 31). Recently, several reports have demonstrated that the qSOFA can accurately assess the severity of community-acquired pneumonia (32–34). However, qSOFA had a lower performance in predicting clinical deterioration compared with other EWS in our study. This may be partially explained by the low percentage of hypotension and alter mental status in this cohort. The finding was also consistent with previous studies, in which qSOFA may not be appropriate to identify critically ill patients with COVID-19 (22, 35).

This study had several limitations. First, this was a retrospective study with relatively small sample size. A larger cohort validation is still required. Second, the changes in EWS during the treatment process was not recorded in this study. Third, as the intubation rate and mortality in this population were lower than that in critically ill patients (25, 35, 36), caution must be taken in extrapolating the results of the study for critically ill patients. Additional assessments of organ dysfunction should be required in critically ill patients with COVID-19.

## Conclusion

The NEWS-C was the most accurate scoring system among common EWS to identify patients with COVID-19 at risk for EDRF and need for IRS. The NEWS-C could be recommended as an early triage tool for COVID-19.

## Data Availability Statement

The original contributions presented in the study are included in the article/Supplementary Materials, further inquiries can be directed to the corresponding author/s.

## Ethics Statement

The studies involving human participants were reviewed and approved by Ethics Commission of Renmin Hospital of Wuhan University. The ethics committee waived the requirement of written informed consent for participation.

## Author Contributions

YS, G-wT, and ZL: conception and design. M-jJ, R-cX, and ZL: administrative support. YS, M-jJ, S-jY, J-lZ, and G-gM: provision of study materials or patients. YS, S-jY, J-lZ, KL, R-cX, and G-gM: collection and assembly of data. YS, G-wT, R-cX, M-jJ, and ZL: data analysis and interpretation. All authors: manuscript writing and final approval of manuscript.

## Conflict of Interest

The authors declare that the research was conducted in the absence of any commercial or financial relationships that could be construed as a potential conflict of interest.
