# Supplementary material for: Prognostic Accuracy of Early Warning Scores for Clinical Deterioration in Patients With COVID-19
Source: Front Med (Lausanne). 2021 Feb 1;7:624255. doi: 10.3389/fmed.2020.624255 (PMC7882600; doi:10.3389/fmed.2020.624255)
Supplement: Supplementary file 1 [file Table_1.DOCX]

**Supplemental Table 1** **Scoring systems of Early Warning Scores.**

The Early Warning Scores were calculated by adding the individual assigned points together. A = alert; CAM, Confusion assessment method (CAM) screening possible for potential delirium; CNS, central nervous system; GCS, Glasgow Coma Scale; P, pain; U, unresponsive; V, voice.

**National Early Warning Score (NEWS)**

| **Physiologic Parameters** | **3** | **2** | **1** | **0** | **1** | **2** | **3** |
| --- | --- | --- | --- | --- | --- | --- | --- |
| Respiratory Rate | <8 |  | 9-11 | 12-20 |  | 21-24 | ≥25 |
| SpO_2_ Scale (%) | ≤91 | 92-93 | 94-95 | ≥96 |  |  |  |
| Air or oxygen? |  | Oxygen |  | Air |  |  |  |
| Systolic Blood Pressure | ≤90 | 91-100 | 101-110 | 111-219 |  |  | ≥220 |
| Heart Rate | ≤40 |  | 41-50 | 51-90 | 91-110 | 111-130 | ≥131 |
| Consciousness |  |  |  | Alert |  |  | VPU |
| Temperature | ≤35.0 |  | 35.1-36.0 | 36.1-38.0 | 38.1-39.0 | ≥39.1 |  |

**New Modified NEWS (NEWS-C)**

| **Physiologic Parameters** | **3** | **2** | **1** | **0** | **1** | **2** | **3** |
| --- | --- | --- | --- | --- | --- | --- | --- |
| Age |  |  |  | <65 |  |  | ≥65 |
| Respiratory Rate | <8 |  | 9-11 | 12-20 |  | 21-24 | ≥25 |
| SpO_2_ Scale (%) | ≤91 | 92-93 | 94-95 | ≥96 |  |  |  |
| Air or oxygen? |  | Oxygen |  | Air |  |  |  |
| Systolic Blood Pressure | ≤90 | 91-100 | 101-110 | 111-219 |  |  | ≥220 |
| Heart Rate | ≤40 |  | 41-50 | 51-90 | 91-110 | 111-130 | ≥131 |
| Consciousness |  |  |  | Alert |  |  | VPU |
| Temperature | ≤35.0 |  | 35.1-36.0 | 36.1-38.0 | 38.1-39.0 | ≥39.1 |  |

**National Early Warning Score 2 (NEWS2)**

| **Physiologic Parameters** | **3** | **2** | **1** | **0** | **1** | **2** | **3** |
| --- | --- | --- | --- | --- | --- | --- | --- |
| Respiratory Rate | <8 |  | 9-11 | 12-20 |  | 21-24 | ≥25 |
| SpO_2_ Scale (%) | ≤83 | 84-85 | 86-87 | 88-92 on oxygen  ≥93 on air | 93-94 on oxygen | 95-96 on oxygen | ≥97 on oxygen |
| Air or oxygen? |  | Oxygen |  | Air |  |  |  |
| Systolic Blood Pressure | ≤90 | 91-100 | 101-110 | 111-219 |  |  | ≥220 |
| Heart Rate | ≤40 |  | 41-50 | 51-90 | 91-110 | 111-130 | ≥131 |
| Consciousness |  |  |  | Alert |  |  | VPU |
| Temperature | ≤35.0 |  | 35.1-36.0 | 36.1-38.0 | 38.1-39.0 | ≥39.1 |  |

**Hamilton Early Warning Score (HEWS)**

| **Physiologic Parameters** | **3** | **2** | **1** | **0** | **1** | **2** | **3** |
| --- | --- | --- | --- | --- | --- | --- | --- |
| Heart Rate |  | ≤40 | 41-50 | 51-100 | 101-110 | 111-130 | >130 |
| Systolic Blood Pressure | <71 | 71-90 |  | 91-170 |  | 171-200 | >200 |
| Temperature | ≤35 |  | 35.1-36 | 36.1-37.9 | 38-39 | ≥39.1 |  |
| Respiratory Rate | <8 | 8-13 |  | 14-20 |  | 21-30 | >30 |
| SpO_2_ Scale (%) | <85 |  | 85-92 | >92 |  |  |  |
| Supplemental Oxygen |  |  |  | Room air | ≤5 L/min |  | >5L/min |
| CNS Change from Baseline |  | CAM |  | A | V | P | U |

**Modified Early Warning Score (MEWS)**

| **Physiologic Parameters** | **3** | **2** | **1** | **0** | **1** | **2** | **3** |
| --- | --- | --- | --- | --- | --- | --- | --- |
| Heart Rate |  | ≤40 | 41-50 | 51-100 | 101-110 | 111-129 | ≥130 |
| Systolic Blood Pressure | ≤70 | 71-80 | 81-100 | 101-199 |  | ≥200 |  |
| Temperature |  | ≤35 |  | 35-38.4 |  | ≥38.5 |  |
| Respiratory Rate |  | <9 |  | 9-14 | 15-20 | 21-29 | ≥30 |
| CNS Change from Baseline |  |  |  | A | V | P | U |

**Quick Sepsis-related Organ Failure Assessment (qSOFA)**

| **Clinical parameters** | **0** | **1** |
| --- | --- | --- |
| Altered mentation | GCS =15 | GCS <15 |
| Respiratory rate | <21 | ≥22 |
| Systolic Blood pressure | >100 | ≤ 100 |
